# Succesful Radiofrequency Ablation of Atrial Tachycardia Arising From Within the Coronary Venous Sinus

**Published:** 2010-05-05

**Authors:** PR Bhima Shankar, TR Muralidharan, S Jaishankar, Gregory Michaud, Narasimhan Calambur

**Affiliations:** 1 Division of Electrophysiology, Department of Cardiology, CARE Hospitals and CARE Foundation, Hyderabad, India; 2 Department of Cardiology, Sri Ramachandra Medical College and Research Institute, Chennai; 3 Cardiac Arrhythmia Section, Cardiovascular Division, Brigham and Women's Hospital, and Harvard Medical School, Boston, Massachusetts

**Keywords:** atrial tachycardia, coronary sinus, radiofrequency ablation

## Background

Focal atrial tachycardia (AT) though a relatively uncommon cause of supraventricular tachycardia is difficult to control with antiarrythmic drugs. With the advent of radiofrequency ablation (RFA), this form of tachycardia can be treated with high long-term success [1]. These foci tend to cluster at specific anatomic locations. In the right atrium, these foci occur along the crista terminalis, the tricuspid annulus, the ostium of the coronary sinus and the perinodal region. In the left atrium, foci occur predominantly at the pulmonary vein ostia and less commonly at the mitral annulus, the left atrial appendage, and the left side of interatrial septum [2-6].

We report the electrophysiological characteristics and ablation procedure of a focal atrial tachycardia which was arising from deep within the coronary sinus.

## Case Report

A 35-year-old male presented with a history of several episodes of palpitation in the preceding 3 years, not controlled by oral metoprolol. The clinical examination was unremarkable. The ECG during sinus rhythm was normal. During tachycardia (rate 220/min), the P wave morphology was difficult to ascertain clearly. Routine biochemical evaluation including thyroid profile was normal. The echocardiogram was normal.

The patient underwent an electrophysiological evaluation under local anesthesia. The procedure was performed with the patient in the fasting, nonsedated state, 48 hours after meteprolol was discontinued. Three 6F quadripolar catheters were introduced to the right atrium (RA), the right ventricular apex (RVA), and at the HB region via the femoral vein. Also, a 7F decapolar catheter (Webster) was advanced within the coronary sinus (CS) via the right internal jugular vein.

**Figure 1:**
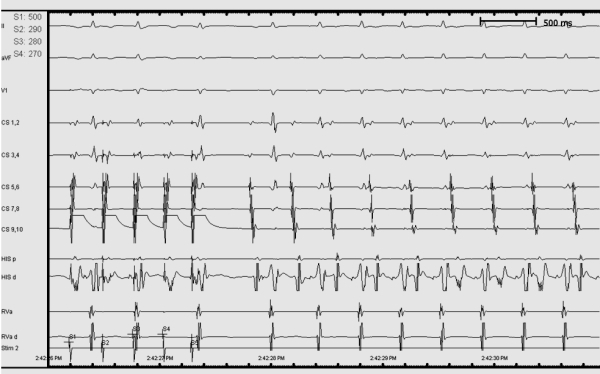
Intracardiac tracing showing induced tachycardia showing varying RP relationship.

Tachycardia was induced with burst pacing from the high right atrium. The induced narrow QRS tachycardia had a cycle length of 330 msec with a variable RP relationship (Figure 1)  The atrial activation sequence was eccentric with the earliest A at the mid CS dipole (CS 3,4). The tachycardia was entrained and the response on cessation of pacing was VAAV.

The posterolateral mitral annulus was mapped with a 7F 4 mm electrode tip cordis Celsius curve type E catheter (Biosense Webster, Diamond Bar, CA). However, the electrograms obtained at that site were later than those recorded from the adjacent electrodes of the CS catheter.

**Figure 2:**
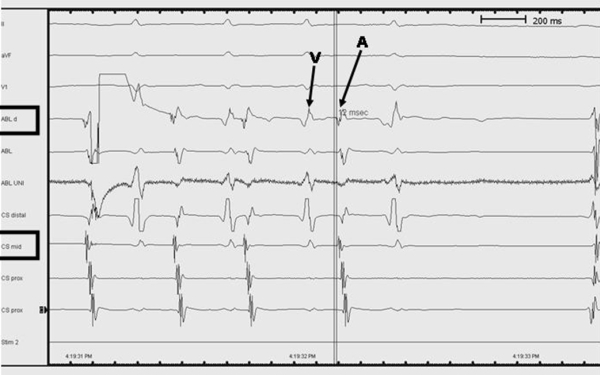
Intracardiac tracings during activation mapping within the CS.

**Figure 3:**
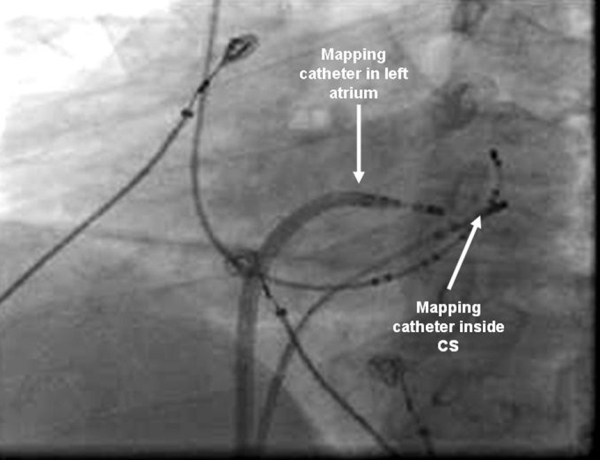
Fluoroscopic (LAO) image showing the mapping catheter within the CS.

The ablation catheter was advanced into the CS from the right femoral vein. Activation mapping within the CS revealed the earliest A around mid CS dipoles. The A in the mapping catheter was 12 milliseconds earlier than in the mid CS dipoles (Figure 2 and 3).

Radiofrequency energy was delivered with continuous temperature feedback control and power output. The target temperature was 50ºC. The tachycardia terminated in 8 seconds and the maximum power delivered was 15 watts. The impedance decreased from 156 ohms to 140 ohms during ablation. Ablation was delivered for 120 seconds. Subsequently, burst pacing at baseline and on isoprenaline did not induce the tachycardia.

## Discussion

Atrial tachycardia arising from within the CS is uncommon. Of the recognized foci of atrial tachycardia origins from CS OS and mitral annulus are less common. Even more uncommon are reports of AT from within CS. Apart from a few isolated case reports the largest  series of AT from within CS is from Badhwar et al who reported a set of 8 patients out of a consecutive series of 283 patients undergoing RFA for AT [7].

The origin of this tachycardia has not been clearly elucidated. It appears to be distinct and seems to arise from CS musculature. Chauvin et al have demonstrated a muscular cuff surrounding the CS extending, on average, 4 cm from ostium. The myocardial sleeve around the CS has been shown to be composed of bands from LA and RA [8].These sleeves are postulated to be the source for these ATs. Another potential source of arrhythmia at the CS is the area of the ligament of Marshall. The ligament of Marshall is a left atrial epicardial neuromuscular bundle that has been associated with the genesis of atrial tachyarrhythmias and atrial fibrillation [9,10].

ATs from within the CS are to be distinguished from ATs from ligament of Marshall (LOM) and mitral annular AT. The need to discriminate ATs that arise in the distal CS from LOM ATs does not arise. However ATs of more proximal origin need to be distinguished. This distinction is pertinent as LOM ATs may require epicardial approach. Posterior mitral annular ATs are the next close differential diagnosis. There have been reports where inadvertent ablation at mitral annulus for CS ATs caused slowing of the tachycardia without termination [7]. In our case we initially mapped the mitral annulus and subsequently within the CS. We obtained the earliest A deep within the CS.

Also a distinct sharp potential preceding the CS A at the site of successful ablation, akin to pulmonary vein potential in pulmonary vein tachycardia has been described in 3 of the case reports and in the five patients of Badhwar series [7]. However we did not obtain such a potential in our patient.

Radiofrequency ablation inside the CS has the inherent problems of ablating inside vascular structures. It was anticipated that attaining adequate power would be an issue considering the catheter tip tissue interface impedance that was high. However on initiating the RF delivery, there was decrease in the impedance and temperature of 50ºC with 30 watts was achieved. In the reports of ablation of AT from CS, conventional radiofrequency ablation were used with success. The safety of ablation within the CS has been shown in the series reported by Irakli Giorgberidze, where left sided accessory pathways were ablated from within the CS [11].

## Conclusions

ATs arising within the CS are uncommon. Mitral annular AT and LOM AT are to be differentiated. It appears safe to deliver conventional energy within the CS although obtaining required power may be a concern due to high catheter tip tissue interface impedance.
